# Why wait? The social determinants underlying tuberculosis diagnostic delay

**DOI:** 10.1371/journal.pone.0185018

**Published:** 2017-09-25

**Authors:** Lily Victoria Bonadonna, Matthew James Saunders, Roberto Zegarra, Carlton Evans, Kei Alegria-Flores, Heinner Guio

**Affiliations:** 1 The University of Michigan, Ann Arbor, Michigan, United States of America; 2 Instituto Nacional de Salud, Lima, Perú; 3 Innovación Por la Salud Y Desarrollo (IPSYD), Asociación Benéfica PRISMA, Lima, Perú; 4 Innovation for Health and Development (IFHAD), Laboratory of Research and Development, Universidad Peruana Cayetano Heredia, Lima, Perú; 5 Infectious Diseases & Immunity, Imperial College London, and Wellcome Trust Imperial College Centre for Global Health Research, London, United Kingdom; 6 Department of Health Policy and Management, University of North Carolina Gillings School of Global Public Health, Chapel Hill, North Carolina, United States of America; Agencia de Salut Publica de Barcelona, SPAIN

## Abstract

**Background:**

Early detection and diagnosis of tuberculosis remain major global priorities for tuberculosis control. Few studies have used a qualitative approach to investigate the social determinants contributing to diagnostic delay and none have compared data collected from individual, community, and health-system levels. We aimed to characterize the social determinants that contribute to diagnostic delay among persons diagnosed with tuberculosis living in resource-constrained settings.

**Methods/Principle findings:**

Data were collected in public health facilities with high tuberculosis incidence in 19 districts of Lima, Peru. Semi-structured interviews with persons diagnosed with tuberculosis (n = 105) and their family members (n = 63) explored health-seeking behaviours, community perceptions of tuberculosis and socio-demographic circumstances. Focus groups (n = 6) were conducted with health personnel (n = 35) working in the National Tuberculosis Program. All interview data were transcribed and analysed using a grounded theory approach. The median delay between symptom onset and the public health facility visit that led to the first positive diagnostic sample was 57 days (interquartile range 28–126). The great majority of persons diagnosed with tuberculosis distrusted the public health system and sought care at public health facilities only after exhausting other options. It was universally agreed that persons diagnosed with tuberculosis faced discrimination by public and health personnel. Self-medication with medicines bought at local pharmacies was reported as the most common initial health-seeking behaviour due to the speed and low-cost of treatment in pharmacies. Most persons diagnosed with tuberculosis initially perceived their illness as a simple virus.

**Conclusions:**

Diagnostic delay was common and prolonged. When individuals reached a threshold of symptom severity, they addressed their health with the least time-consuming, most economically feasible, and well-known healthcare option available to them. In high-burden settings, more human and material resources are required to promote tuberculosis case-finding initiatives, reduce tuberculosis associated stigma and address the social determinants underlying diagnostic delay.

## Introduction

Although effective treatment has existed for more than 50 years, tuberculosis (TB) is the most frequent infectious cause of death worldwide [1]. An estimated 10.4 million people developed TB disease in 2015 –the majority of cases occurring in low- and middle-income countries such as Peru [1]. Although Peru has a model National TB Program (NTP) that incorporates directly observed therapy (DOT) and provides medications, diagnostic tests, and psycho-social services free of direct charges, it continues to have the second highest TB burden in the Americas. In 2015, the estimated incidence rate was 119 per 100,000 population [2]. The incidence of multi-drug resistant (MDR) TB is the highest of all American countries and continues to increase [2,3].

Early detection, diagnosis and notification of TB is a global priority for TB control efforts and is emphasized in the World Health Organization (WHO) End-TB Strategy [4, 5]. In 2015, only 57% of TB cases were estimated to have been diagnosed and notified globally [1]. Low case detection and delays in treatment initiation contribute to increased household and community transmission of TB, severity of individual disease, and risk of adverse treatment outcomes [6].

Globally, TB is recognized as a socially stratified disease that clusters among disadvantaged groups. This has recently been confirmed in the setting of the current research [7,8]. An inequitable distribution of low income, food insecurity, poor housing, unhealthy environmental conditions, and other social determinants exacerbate poor TB control in areas of low socio-economic position [9]. In order to meet the ambitious targets set out in the End-TB Strategy and ensure the prompt diagnosis and treatment of often vulnerable individuals, it is important to understand the relationships between social determinants and TB diagnostic delay [10], and how interventions may address these social determinants [11].

The reasons and risk factors for diagnostic delay have been investigated in many parts of the world and vary considerably according to the context [12–18]. Several studies have examined diagnostic delay, but none have used comparative analyses of qualitative data collected from persons diagnosed with TB (PDTB), family members, and health personnel working in the NTP [19–21].

The objective of this study was to identify and characterize the social determinants that contribute to diagnostic delay among PDTB living in Lima and Callao, Peru. We focused on the perspectives of PDTB, their family members, and health personnel in order to gain an individual, community, and health-system level understanding of this phenomenon.

## Methods

### Setting

From May to October 2015, this study was conducted in 25 public health facilities throughout 19 districts of Lima and Callao, Peru. The estimated population of Lima and Callao is 9.8 million people living in 50 districts, defined by having a mayor and municipality. In order to select the districts for this research, we met with regional TB coordinators in 7 health management network offices to identify geographically distinct districts with the highest respective TB burdens for their network. Districts varied from desert shantytowns to dense, historical city neighbourhoods. All public health facilities had a designated TB treatment program with at least one nurse technician devoted exclusively to working with PDTB.

### Participants

Inclusion criteria were PDTB (n = 105) aged at least 18 years old, who were currently taking treatment for their first episode of pulmonary TB in a public health facility regulated by the NTP, whose NTP treatment card indicated that they had had a sputum smear microscopy positive result. In Peru, sputum smear microscopy is the only widely-used rapid laboratory test. Culture and molecular testing are largely restricted to drug-susceptibility testing after TB has been diagnosed. We included people with drug-sensitive and drug-resistant TB. Exclusion criteria were inability or unwillingness to give written, informed consent, and history of past TB disease.

PDTB were selected to participate by convenience sampling whereby we approached individuals taking treatment for TB at selected public health facilities who happened to be present at the time of our visit. Approximately 10% of PDTB declined to participate in our study. All recruited PDTB gave written informed consent to be interviewed and were asked to agree to a home visit. Family members interviewed (n = 63) were currently living in the same household or city district as the PDTB. Recruited family members were also selected by convenience sampling whereby we approached them at the time of our home visits. Six focus groups were held, each with 5–8 health personnel. These health personnel included doctors, nurses, nutritionists, lab technicians, social workers, nursing technicians, and/or psychologists who currently and/or previously worked with PDTB in the study districts. Family members and health personnel gave verbal consent to participate.

### Data collection

Healthcare experiences and community perceptions of TB disease were explored with current PDTB, their family members, and health personnel who currently and/or previously practiced in the NTP. Two methods were used: (1) semi-structured interviews; and (2) focus group discussions.

#### (1) Semi-structured interviews

All PDTB were interviewed using a semi-structured interview guide developed from a prior WHO survey of diagnostic and treatment delay [12]. This document was reviewed and edited by three Peruvian medical professionals with more than twenty years of experience with TB in-country. Thereafter, it was piloted during 2 pilot interviews with PDTB and modified based on their responses. The pilot interviews were only used for refining the interview guide, were not recorded, and are not further reported here. Data from pilot interviews are not included in the main study. The main study interviews lasted 30 minutes to 1 hour, over 1 or 2 meeting occasions, and explored the following themes: socio-demographics; household environment and family composition; migration history; past medical history; history of patient’s illness including symptoms; health-care seeking behaviours; and socio-economic consequences of TB. Medical charts were also reviewed for history of past TB disease and other comorbidities. Interviews with family members lasted 5 to 30 minutes and were conducted either in the public health facility or the family member’s home, depending on their preference. These interviews focused on community perceptions of TB disease, its causes and social consequences, common health seeking behaviours and interactions people had with the public health system. All interviews were conducted in Spanish and digitally recorded. These recordings were then transcribed for analysis except in three cases when recording was declined. Detailed notes were taken in these cases.

#### (2) Focus groups

Focus groups with health personnel lasted 40 to 90 minutes and questioned community perceptions of TB disease and common health seeking behaviours. Relationships and attitudes between health personnel, PDTB, and the NTP were discussed. Participants were encouraged to provide what they thought were the general opinions and behaviours of the populations in which they worked. The discussions were freeform, such that participants could respond to one another’s comments and build on emerging themes. Interview guides for PDTB, family members, and health personnel are shown in the online supplement.

**Analysis**. Diagnostic delay was defined as the number of days between symptom onset and the first smear positive TB diagnostic sample recorded by public health facilities regulated under the NTP. PDTB first described their symptoms of TB and then we specifically asked them if they had experienced cough, fever, weight loss, and/or hemoptysis. Symptoms described by PDTB included cough, fever, weight loss, hemoptysis, fatigue, and night sweats. We used the date PDTB self-reported their first symptom related to TB as the date of symptom onset. The number of days of diagnostic delay was then back-calculated using this date.

All spoken words from the interviews and focus groups were analysed using a grounded theory approach. Interviews and focus groups were reviewed to identify emerging themes and concepts. Using the software Atlas.ti 7 (Scientific Software Development GmbH, Berlin, Germany) a list of conceptual codes was then created to organize and group information provided by participants. A fully coded analysis of all transcripts was completed and used to create a conceptual framework of factors contributing to diagnostic delay. Translation of representative quotes from Spanish to English was done after analysis. Descriptive statistics were calculated using Excel 2013 (Microsoft Office).

**Ethics statement**. The study received ethical approval from the Institutional Committee of Research Ethics at the Peruvian National Institute of Health and the Internal Review Board of Health Sciences and Behavioral Sciences at the University of Michigan, USA.

## Results

Demographic characteristics of all PDTB are shown in Table 1. The median age was 28 and 38% were female. Table 2 shows characteristics of the family members interviewed. The median age was 46 and 87% were female. Table 3 outlines characteristics of the focus groups, for whom the median duration working with PDTB was five years.

**Table 1:** Demographic characteristics of all persons diagnosed with TB.

	Variable	Units	Persons diagnosed with TB (n = 105)
**General**	Age	median (IQR)	28 (22–49)
	Female sex	n (%)	40 (38)
	Monthly personal income	median $US (IQR)	218 (116–349)
**City districts**	San Juan de Lurigancho	n (%)	18 (17)
	El Augustino	n (%)	7 (7)
	La Victoria	n (%)	14 (13)
	Santa Anita	n (%)	6 (6)
	ATE	n (%)	2 (2)
	Lurin	n (%)	3 (3)
	Villa Maria del Triunfo	n (%)	3 (3)
	Villa El Salvador	n (%)	3 (3)
	San Juan de Miraflores	n (%)	4 (4)
	Bellavista	n (%)	3 (3)
	Rimac	n (%)	5 (5)
	San Martin de Porres	n (%)	14 (13)
	Independencia	n (%)	3 (3)
	Los Olivos	n (%)	6 (6)
	Callao	n (%)	3 (3)
	Ventanilla	n (%)	4 (4)
	Mi Peru	n (%)	1 (1)
	Carabayllo	n (%)	3 (3)
	Comas	n (%)	3 (3)

**Table 2:** Demographic characteristics of all family members interviewed.

	Variable	Units	Family member (n = 63)
**General**	Age	median (IQR)	46 (39–54)
	Female sex	n (%)	55 (87)
	Previously had TB	n (%)	3 (5)
**Relation to person diagnosed with TB**	Parent	n (%)	31 (49)
	Sibling	n (%)	5 (8)
	Child	n (%)	6 (10)
	Spouse	n (%)	10 (16)
	Other	n (%)	11 (17)

**Table 3:** Demographic characteristics of focus groups.

	Variable	Units	Focus Group Discussion (n = 6)
**General**	Total health professionals	n (%)	35 (100)
	Number of health professionals per focus group	median (IQR)	5.5 (5–6)
	Years working in TB	median (IQR)	5 (3–14.5)
**Occupations**	Physician	n (%)	11 (31)
	Technical nurse	n (%)	7 (20)
	Nurse	n (%)	12 (34)
	Nutritionist	n (%)	2 (6)
	Laboratory technician	n (%)	1 (3)
	Psychologist	n (%)	1 (3)
	Social worker	n (%)	1 (3)

The age of health personnel was not recorded for confidentiality purposes. Note: IQR indicates interquartile range; TB indicates tuberculosis.

### Diagnostic delay

**Fig 1:**
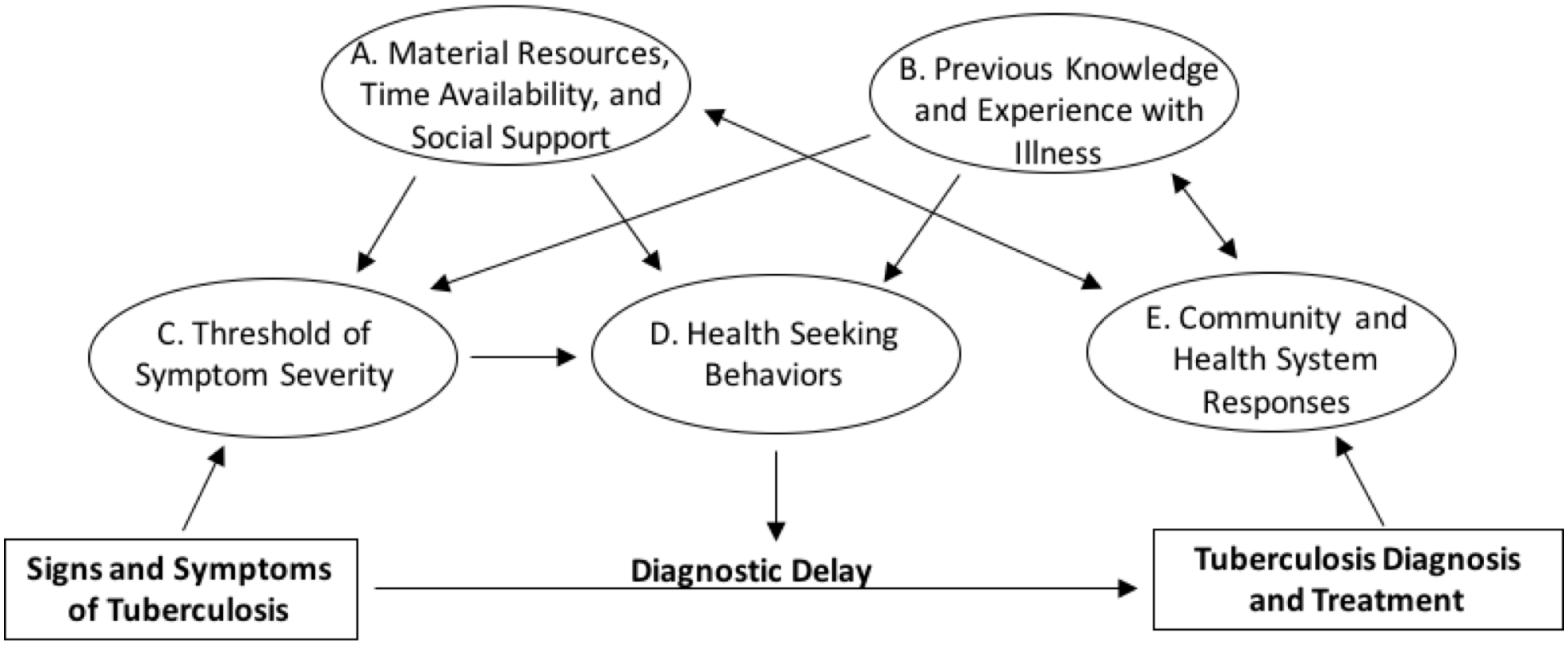
Conceptual framework of factors contributing to diagnostic delay. See the results section of the manuscript for explanations of categories A, B, C, D, and E.

The median diagnostic delay was 57 days (interquartile range (IQR): 28–126). Interview and focus group data were organized conceptually into five categories that directly or indirectly contributed to diagnostic delay. Categories are illustrated in Fig 1 and are presented below with direct quotes from PDTB, family members, and health personnel. Table 4 shows descriptions of codes used to create the conceptual framework.

**Table 4:** Codes used to create the conceptual framework.

Factors contributing to diagnostic delay	Conceptual codes: PDTB	Conceptual codes: family members	Conceptual codes: health personnel
**A. Material resources and social support available**	Initial reactions to symptoms	Reasons for high TB prevalence	Reasons for high TB prevalence
	Knowledge of TB prior to diagnosis	Groups at greater risk of contracting TB	Groups at greater risk of contracting TB
	Knowledge of TB transmission	Common community knowledge of TB	Common community knowledge of TB
	Knowledge of causes of TB		Initial reactions to TB diagnosis
	Other health beliefs related to TB mentioned		Generalized beliefs and behaviors of PDTB
	Reasons why individuals wait to seek care at public health posts		
	Initial reactions to TB diagnosis		
**B. Previous knowledge and experience with illness**	Knowledge of TB transmission	Reasons for high TB prevalence	Reasons for high TB prevalence
	Knowledge of causes of TB	Groups at greater risk of contracting TB	Common community knowledge of TB
	Other health beliefs related to TB mentioned	Common community knowledge of TB	Initial reactions to TB diagnosis
	Reasons why individuals wait to seek care at public health posts		
	Initial reactions to TB diagnosis		Initial reactions to TB diagnosis
**C. Threshold of symptom severity**	Reasons for seeking professional medical attention	Health seeking behaviors	Average time PDTB wait before they seek attention at a public health facility
	Progression of symptoms to diagnosis	Health seeking behaviors following hemoptysis	
**D. Health seeking behaviors**	Health seeking behaviors	Health seeking behaviors	Practices of private clinics
	Reasons for seeking attention from a professional health facility	Motivations for self-medication	Motivations for self-medication
	Description of first experience with a professional health facility		
	Time waiting at first professional health facility		
	Personal treatment received from first professional health facility		
**E. Community and health system reactions to TB disease**	Personal treatment received from first professional health facility	Community opinions of TB	Attitudes of health personnel working in the National Tuberculosis Program
	Concealment of TB diagnosis	Community treatment of PDTB	Interactions between health personnel and PDTB in the public health post
	Changes in work or studies due to TB diagnosis		Consequences of TB diagnosis
	Embarrassment due to TB diagnosis		Political inhibitions to TB control
	Changes in familial interactions due to TB diagnosis		Reasons for high TB prevalence
	Changes in social interactions due to TB diagnosis		Groups at greater risk of contracting TB

Note TB indicates tuberculosis; PDTB indicates persons diagnosed with TB.

#### A. Material resources, time availability, and available social support

The median individual income of all PDTB was US $218 per month (IQR: 116–349). It was universally agreed that indirect and direct costs associated with healthcare visits often contributed to patients’ delay in seeking professional care.

“[I waited] because of work…there was no time. I returned in the night from my job, around 8:30 pm and at this time the health posts are not open.”

(Male Patient, Interview)

“I didn’t want to bother my children. I felt so alone. I didn’t want to leave [my house]. My husband treated me bad and I wanted to die…I didn’t have money [to go to the doctor].”

(Female Patient, Interview)

For those PDTB who did have available social support, preoccupation by family and friends was noted as a key motivation for accessing health facilities.

“In the month of April I started to feel a little sick and I was coughing frequently and my mom got worried and made me go to the hospital for an appointment.”

(Female Patient, Interview)

Fear associated with a possible diagnosis was frequently mentioned as another barrier to seeking professional medical attention.

“Some people are afraid of what they [the doctors] will say. They have TB and they [the patients] think that the people are going to treat them poorly.”

(Family Member, Interview)

Health personnel also mentioned that fear of being sick extended to fear of job loss. As discussed in every focus group, PDTB often struggled to maintain sufficient household income in resource-constrained settings.

“For example, in [their neighbourhoods], they live in completely overcrowded conditions not because they want to, but because their economic situation obligates them to be there.”

(Health Care Worker, Focus Group)

#### B. Previous knowledge and experience with illness

Prior to diagnosis, almost all PDTB had, at least, heard of the existence of a disease called TB in their communities. PDTB generally associated TB with poor nutrition and low immune defences due to excess of work, stress, or a “disordered” life of drug, alcohol, and cigarette use for example.

“I thought TB was for people who did not eat, for the vagabonds that didn’t look after their things.”

(Male Patient, Interview)

When PDTB first started experiencing typical TB symptoms, it was very often attributed to a common virus.

“I sincerely thought it was something that would pass because I never would have thought to get this disease. I thought it was a cough.…”

(Male Patient, Interview)

Family members held similar ideas of TB as a nutritional disease that predominantly affects those with weak immune systems.

“Weak people [are at major risk]. For example, a person dedicated to studying in the university. He, [my son] got good grades in school and he neglected food…and the bus dropped him very far from the university. All this physical activity and responsibility killed my son.”

(Family Member, Interview)

Community knowledge of TB treatment was variable; some family members reporting widespread knowledge of Peru’s NTP and others none.

“I guess that, if there was a percentage, 70% don’t know [about treatment for TB].”

(Family Member, Interview)

“The whole world knows that if you have TB you leave your sputum for free and they give you your medicine for free.”

(Family Member, Interview)

Health personnel agreed that upon seeing PDTB for the first time, many have some awareness of the disease, although few know details such as treatment length or the quantity or type of treatment medications.

“When they come for the first time, they tell us it’s a contagious disease. And they come worried because maybe they have infected someone.… So they have some knowledge, but what they don’t know is how long treatment lasts. They only know it is contagious.”

(Health Care Worker, Focus Group)

#### C. Threshold of symptom severity

Eventually, every PDTB’s health deteriorated to a point where they had to seek medical attention. Although each PDTB held their own individual threshold, most went to see a physician after ill health prevented them from conducting daily activities including working; was noted as extremely abnormal; or persisted for an unusually long time.

“I lost weight because I was sweating a lot. I felt really cold and I lost 15 kilos in two weeks and this was very worrisome.”

(Female Patient, Interview)

“The cough made my chest hurt. I couldn’t take it…I started to feel pain behind in my back. I had fever and my body hurt. My fever was too high.”

(Male Patient, Interview)

“It came to the extreme…I threw up blood from my nose, my mouth…and I went to the hospital.”

(Male Patient, Interview)

Half of family members agreed that most people do not seek medical attention until a relatively high threshold of symptoms is passed, such as when people begin to cough blood.

“Here in Peru people wait to see if it will pass normally and when they fall really sick that is when they go [to the doctor].”

(Family member, Interview)

“[Coughing blood] is something else. Then they go to the hospital. Definitely the hospital.”

(Family Member, Interview)

In focus groups, health personnel noted that most PDTB normally waited weeks, if not months, to seek treatment, after symptoms appeared.

“Some delay, some wait, coughing, and they come when it doesn’t pass with any pills and they start to vomit blood….”

(Health Care Worker, Focus Group)

#### D. Health seeking behaviours

PDTB first health seeking behaviours were largely determined by influential factors described in the three previous sections. The most common initial behaviour, was to self-medicate with pills including antibiotics such as amoxicillin and levofloxacin, cough syrups, injections bought without medical prescription from pharmacies or natural medicines.

“It was for a throat infection. Every day I took different pills because I was going to different pharmacies and they told me I was sick in the throat.… I thought it would pass in the night and the next day it came back.… At this time I didn’t have health insurance and I didn’t have time [to go to the doctor].”

(Female Patient, Interview)

Unlike public health facilities, most pharmacies stay open until late in the night, after working hours. They are the most accessible facilities to receive healthcare, especially for working people who leave their homes early in the morning and return in the night. Medical advice provided by the individuals working there is also free and rapid and does not require proof of health insurance.

“They always go to the pharmacy and they say this or that and they get a diagnosis and a pill. It’s faster…sometimes there is no time to go to the health post…Here you have to work and if you don’t, you don’t survive.”

(Family Member, Interview)

However, as noted in focus groups, the advice given in pharmacies very rarely, if ever, is given by professionally trailed health practitioners. Individuals cannot receive the correct tests or treatment for TB so attention provided by pharmacies may increase diagnostic delay in these communities.

“In [the pharmacy] it is not the pharmacist that sees you, but rather the technical assistant, but not even the pharmacist is authorized to prescribe [TB] medications.”

(Health Care Worker, Focus Group)

“I have seen [the pharmacy] give levofloxacin for up to three days. Imagine that.”

(Health Care Worker, Focus Group)

Using natural medicine was another common health seeking behaviour before seeking care from a physician.

“[I took] coca caramels, water with salt, some herbs, but nothing passed…Pills are a lot of chemicals and they told me that first you start with natural things and then take drugs…”

(Female Patient, Interview)

The remaining PDTB who did not first self-medicate or take natural medicine used public health facilities, public hospitals, employer insured health facilities, or private clinics as their first health seeking behaviour. Their decision to pay out-of-pocket expenses for private care or use the state-insured or employer-insured health system largely depended on the person’s amount of available time and money and where they held the greatest trust. Long wait times were frequently noted as an obstacle for using the insured systems. Health personnel admitted that many individuals resorted to use of these facilities only if it was the last available option for them.

“People have the perception that if they go to a private doctor they are going to receive better attention…”

(Health Care Worker, Focus Group)

“The majority come with one month of symptoms—they thought it was something normal or they went to the pharmacies or did what their mother, sister, grandmother told them to do. When the symptoms persist they go to a private professional and since he doesn’t have much experience he says upper respiratory infection. And then they continue hurting and then finally go to the public health post when a lot of time has passed.”

(Health Care Worker, Focus Group)

Although all PDTB eventually did seek professional medical attention, most had to visit two or more professional health facilities to receive a diagnosis of TB and enter the NTP to receive free treatment.

“I went to the [private] doctor and he gave me shots, cough syrups, and it didn’t pass. The fever went, the body aches went, but the cough didn’t…. Again, I went to the [private] doctor and he gave me another cough syrup and a pill, an antibiotic…Nothing passed…A woman [in my neighbourhood] told me to go to a public health clinic close by so I could leave my sputum sample because the cough is dangerous and maybe I have TB…”

(Male Patient, Interview)

#### E. Community and health system response to TB disease

After diagnosis, the great majority of PDTB reported that they faced discrimination within their communities. Most admitted hiding their diagnosis from friends, work colleagues, and even some family members, attempting to avoid stigma and its subsequent socio-economic consequences.

“I don’t want them to be scared.… I hide [my diagnosis] for work because if I didn’t, I wouldn’t be working…”

(Male Patient, Interview)

“Sometimes people think that TB is for people who have bad lives, but in my case, I don’t have a bad life because I don’t drink, I don’t smoke, I don’t go to parties…”

(Female Patient, Interview)

“If you have TB…you feel like a source of infection and the people are going to, they are going to reject you…”

(Male Patient, Interview)

Family members agreed that discrimination within communities often stemmed from a fear of disease contagion.

“They are scared, they say it’s contagious and they don’t want to go close to [the TB patient]. They isolate them.”

(Family Member, Interview)

Health personnel admitted, in concordance with testimonials from PDTB, that discrimination within health facilities was strong and common.

“I had a patient and I asked him, why did you abandon treatment? He said the nurse left him the pills on the table and then everyone disappeared, everyone left running.… The patient said: why am I going to come here if they treat me bad, if I feel bad? A TB patient has low self-esteem, and with the treatment we give him it gets worse….”

*(Health Care Worker, Focus Group)*.

Health personnel in focus group discussions often noted that many of their colleagues did not want to work in the NTP for fear of contagion.

“There are some who consider it a punishment and look for a way to get out of working in the TB Program.”

(Health Care Worker, Focus Group)

As noted in focus groups, although the Peruvian NTP has established treatment guidelines, many of them are not strictly followed due to a lack of human resources. Similarly, NTP policies state that all PDTB should receive support from several different health professionals including psychologists throughout treatment, but public health facilities often cannot complete the array of specialty appointments the NTP aims to provide.

“This is the route: nurse, doctor, psychologist, nutritionist, social worker, and obstetrician if you are a woman. This is the route, but it’s not really like this. We don’t follow it because there are not complete teams in the health posts, and in those that are they can’t meet the demand…”

(Health Care Work, Focus Group)

The lack of political will to support a stronger multi-disciplinary effort against TB was also described during focus groups as a major barrier to finding disease and subsequently decreasing diagnostic delay in high burden areas. With limited health staff, public health facilities are often left with no resources to run social and/or educational programs that might reduce stigma, disseminate information into communities and/or encourage prospective patients to seek care at public health facilities.

“There is a multi-sectorial strategic plan and it involves the ministry [of health], but it is not taken up. They do not give it the importance it should be given.… [It] is a plan that should include all of the ministries, but it is not being applied…”

(Health Care Worker, Focus Group)

“We have technical guidelines, which obligate that we focus on integral treatment, and they also include an educational part and require private and public institutions to make work plans [for PDTB] that they are still not executing. We should work so that people…have more information [about TB] and break stigmas that TB is only for poor people, homeless people, for ignorant people…We have to [advertise our program] as free. Some people don’t come because of the cost. They are scared of [losing] money.”

(Health Care Worker, Focus Group)

## Discussion

Prompt diagnosis and treatment of TB is important for reducing morbidity, mortality, and preventing TB transmission [22,23]. Our results show that PDTB generally experienced symptoms associated with TB for approximately two months before receiving a diagnosis. These significant diagnostic delays were largely influenced by five defining areas: (A) material resources, time availability, and available social support; (B) knowledge and previous experience with illness; (C) threshold of symptom severity; (D) health seeking behaviours; and (E) community and health system responses to TB disease. Understanding these five areas may help strengthen health systems seeking to improve case-finding and health promotion strategies. Consequently, this may reduce detrimental diagnostic delays [24–27] that increase community transmission, a major contributor to high TB incidence rates [28].

During focus groups and interviews, many individuals associated TB with poor nutrition and high consumption of alcohol and/or illegal drugs, as reported previously in Peru [29]. This belief created a stereotypical profile of PDTB within communities and may subsequently contribute to diagnostic delays for individuals who have symptoms of TB if they do not suspect that they have TB because they do not fit this stereotype. Although only a small proportion of PDTB in our sample reported extensive use of alcohol and/or illegal substances, our data is subject to recall and social desirability biases. Despite this, our results suggest that the association of TB with substance abuse was exaggerated within communities and by health personnel in our setting. This exaggeration may contribute to the stigma felt by PDTB and by extension, the reported fear that delayed some individuals from seeking healthcare. Many PDTB associated their symptoms to a common virus and as in other studies, lack of knowledge of TB was associated with diagnostic delay [30,31]. Our findings suggest that grade of knowledge influences threshold of symptom severity and health seeking behaviours by PDTB, as shown in Fig 1.

As reported in other studies, many PDTB prioritized working over seeking professional medical attention when they began to feel ill [31,32]. The income of most participants was similar to Peru’s minimum monthly salary of US $215 and although TB diagnostic tests are free of direct charges in Peru, many PDTB reported anticipated costs associated with visiting a physician as a reason for diagnostic delay [33]. Furthermore, wait times were perceived as extremely long when using public or employer-insured health facilities, as was quantified in another study in Peru [31] and described in other settings [15]. If PDTB had the economic resources to do so, many preferred to first visit private clinics, a preference previously reported by other studies [32,34]. We suggest that the Peruvian NTP collaborates with private clinics to promote consideration of TB as a possible diagnosis amongst their patients and additionally, encourage all PDTB to seek free treatment in public and employer-insured health facilities as quickly as possible.

Overwhelmingly, PDTB in this study declared that they had little trust in the public health system. Most PDTB admitted to visiting public health facilities only when they continued to experience symptoms and their other options for available healthcare decreased. As in other studies, when economically insecure, time-pressured individuals enter a resource-constrained public health system, we found that most PDTB experienced stigma [35–37]. The shared demand for material resources and social and occupational support amongst actors in the health system may maintain community knowledge gaps, weaken trust and entrench already established stigma. This may contribute to diagnostic delay through the relationships shown between areas (A), (B), and (E) of Fig 1. Studies from Malawi, Nepal, and Ghana have shown stigma produced through similar relationships between PDTB and health personnel [15,38,39].

Statements by PDTB, family members, and health personnel suggested that strong social support acted as a catalyst for seeking care in professional health facilities. This supports similar qualitative results from the Philippines which describe the importance of accompaniment in initiating care seeking behaviour [32]. Additionally, a study from Uganda found that individuals most often seek advice from their immediate social network when first experiencing symptoms of TB [40]. In our study, a lack of social support may then contribute to diagnostic delay through increasing the threshold of symptom severity required before initiating health seeking behaviours. Consequentially, these health seeking behaviours may lead to further delay based on the time to diagnosis in public health facilities.

Self-medication was frequently reported when PDTB first began to experience TB symptoms. Self-medicating with antibiotics and other medications bought from pharmacies in areas including Tanzania, has been shown to contribute to diagnostic delay and may also contribute to the generation of antibiotic resistance [41–44]. The high proportion of PDTB attending pharmacies before visiting professional health facilities identifies a location that future public health campaigns could target in their TB case-finding initiatives. Globally, few pharmacies advise their customers to seek professional medical attention [34,45,46]. We suggest that the Peruvian NTP collaborate with pharmacies to train their employees to identify customers seeking medications to relieve symptoms of possible TB. We encourage the NTP to offer these pharmacies information leaflets and sample containers that customers, or the individuals for whom they represent, could deliver to the closest public health post for free TB testing. This working relationship between the NTP and pharmacies could be established through recovering PDTB, who may be recruited to help find TB in pharmacies operating in the neighbourhoods in which they live.

This study has several limitations. Reporting and recall biases may have occurred because participants provided responses retrospectively. We aimed to reduce this by questioning and reconfirming participants’ narratives. The study is also subject to selection bias because we used convenience sampling to recruit PDTB and their family members. However, the study took place in multiple diverse health centres and approximately 10% of patients declined to participate, so any possible selection biases could not have explained our findings. Additionally, we only studied diagnostic delay in adults, and did not include children and adolescents who are high risk groups for TB. The study design inevitably excluded people with undiagnosed TB because they were either: (1) people with never-to-be-diagnosed prevalent TB that can only be diagnosed by door-to-door prevalence surveys; or (2) chronic and/or paucisymptomatic TB that had not yet been diagnosed. Another limitation concerns our definition of diagnostic delay as the number of days between symptom onset and the first smear positive TB diagnostic sample recorded by public health facilities regulated by the NTP. This definition may neglect some earlier sputum samples that could have been positive for TB if more sensitive tests like sputum culture had been used. The precise number and characteristics of PDTB who declined to participate were not recorded; however, this affected approximately 10% of PDTB and so it is unlikely to have caused important bias. This issue has prompted current ongoing research interviewing PDTB who decline research participation, to better understand how their characteristics may differ from research participants.

The first author of this manuscript was the only person who fully coded transcripts for qualitative data analysis. In order to ensure high-validity of research results, we triangulated sources and methods by conducting interviews with patients and their family members and focus groups with health personnel. All data were analysed using a grounded theory approach, whereby the person carrying out analysis remained aware of her own preconceived ideas and used “bracketing” to prevent these ideas from influencing her examination of accounts from participants [47]. Data were collected past the point of saturation, after no new ideas emerged from interviews or focus groups.

Future research might quantify subsequent health system delay following TB diagnosis. This is the time between official diagnoses in the NTP and start of treatment. Although other studies have noted this delay to be comparatively minor, further research may shed light on additional opportunities to strengthen Peruvian NTP activities and help pinpoint other needed resources [22]. Future research should also evaluate social and/or economic interventions to reduce diagnostic delay with interventions that may be informed by our findings.

## Conclusions

Overall, this study demonstrated that diagnostic delays are frequent, and associated with important underlying social determinants. PDTB, their family members and health personnel all stated that when individuals reach a threshold of symptom severity, they address their health with the least time-consuming, most economically feasible and well-known healthcare option available to them. Our findings suggest that in Peru and other resource-constrained settings the development and/or improvement of policies that address and help reduce diagnostic delays are urgently needed to reduce community transmission, morbidity, and mortality. Case-finding initiatives should target locations that are a first point of contact for possible PDTB, shown to be pharmacies in our research. More and improved material and human resources are required in health clinics and private sector health facilities to implement these strategies, reduce TB associated stigma, and address the multifaceted social determinants of TB.

## Supporting information

S1 Appendix Interview guides for persons affected by TB, family members, and focus groups with health personnel. (PDF)
