# Supplementary material for: Why wait? The social determinants underlying tuberculosis diagnostic delay
Source: PLoS One. 2017 Sep 25;12(9):e0185018. doi: 10.1371/journal.pone.0185018 (PMC5612650; doi:10.1371/journal.pone.0185018)
Supplement: S1 Appendix — (PDF) [file pone.0185018.s001.pdf]

I. Datos Generales

Fecha de la entrevista (dd/mm/aaa): _____/_____/_____
Hora de inicio de entrevista: ____
Lugar de la entrevista: ____
Dirección de Salud: ____
Red de Salud: ____
Establecimiento de salud: ____

II. Características sociodemográficas, vivienda y factores de riesgo

Fecha de Nacimiento (dd/mm/aaa): _____/_____/_____

Lugar de Nacimiento:
Provincia: ____
Departamento: ____
Distrito: ____

Distrito de residencia: ____

Tiempo de residencia: 1. Menor a 1 año ☐ 2. De 1 a 5 años ☐ 3.Mayor a 5 años ☐

Grupo étnico: Mestizo ☐ Blanco/a ☐ Negro/a ☐ Asiático/a ☐

Grado de instrucción:

1. Sin nivel ☐
2. Primaria completa ☐
3. Primaria incompleta ☐ Especificar: 1☐ 2☐ 3☐4☐5☐6☐7☐
4. Secundaria completa ☐
5. Secundaria incompleta ☐ Especificar: 1☐ 2☐ 3☐4☐5☐6☐7☐
6. Sup. Técnico completo ☐
7. Sup. Técnico incompleto ☐ Especificar: 1☐ 2☐ 3☐4☐5☐6☐7☐
8. Sup. Universitario complete ☐
9. Sup. Universitario incompleto ☐ Especificar: 1☐ 2☐ 3☐4☐5☐6☐7☐

¿Cuáles fueron algunos de los estímulos/ inhibiciones para que Ud. acceda a educación superior?
____
____
____

Estado Civil:

1. Soltero (a) ☐
2. Casado(a) ☐
3. Conviviente ☐
4. Separado ☐
5. Divorciado(a) ☐
6. Viudo(a) ☐

Numero de hijos: 1☐ 2☐ 3☐4☐5☐6☐7☐
Edad(s): ____

Tipo de vivienda:

1. Partícula ☐ 1.1.Casa independiente ☐
   1.2.Departamento ☐

2. Colectiva ☐ 2.1.Vivienda en quinta ☐
   2.2.Vivienda en casa de vecindad ☐

3. Sin Vivienda ☐

Tenencia de la vivienda:

1. Propia ☐
2. Alquilada ☐
3. Vivienda familiar ☐ Especificar: ______________________
4. Alojado ☐
5. En la calle ☐

Características de la vivienda.
Puedes describir su casa? Por ejemplo, el material que hacen la casa, la disponibilidad de agua en la casa, y el tamaño de la casa.

1. Material predominante en las paredes: ____
2. Material predominante en los pisos: ____
3. Material predominante en los techos: ____
4. Abastecimiento de agua: ____

Número de habitaciones: 1☐ 2☐ 3☐4☐5☐ Número de dormitorios: 1☐ 2☐ 3☐4☐5☐

Ud. Comparte su dormitorio con algún otro miembro de la familia? Sí ☐ No ☐

Hábitos Nocivos.
Ud. ¿Ha fumado cigarrillos? Sí ☐ No☐ Actualmente ☐
Numero de cigarrillos al día: __________________
¿Por cuantos años? __________________________

Ud. ¿Ha consumido alcohol? Sí ☐ No ☐
¿Cuantas veces por semana? 1☐ 2 ☐ 3☐ 4☐5☐ 6☐7☐
¿Cuantas copas? 1☐ 2 ☐ 3☐ 4☐5☐ 6☐7☐ 8☐ 9 ☐ 10☐

Ud. ¿ha consumido drogas?
Sí ☐ No ☐ Actualmente ☐ ¿Por cuantos años? _______________
Pasado ☐ ¿Por cuantos años? ______________________________
¿Qué tipo?____

III. Composición familiar

Número de miembros de la familia con los que vive: ________________

Nº	EDAD	SEXO	PARENTESCO	TRABAJA		ACTIVIDAD (Descripción de Trabajo)	Grado de INSTRUCCIÓN	APORTE ECONÓMICO		CANTIDAD QUE APORTA	¿Tiene Tuberculosis?	
				SI	NO			SI	NO		SI	NO
1												
2												
3												
4												
5												
6												
7												
8												
9												

IV. Características Socioeconómicas

¿Ud. se encuentra trabajando actualmente? Si ☐ 2. No ☐
*Si está definido por más de 8 horas cada día.

De ser afirmativa señale el trabajo que realiza: (Titulo y descripción)
____
____

¿Cuáles son sus horas de trabajo normal?
____
____

¿Cómo es su entorno de trabajo?
Luz artificial ☐
Luz natural ☐
Lleno de gente ☐
Tiene ventanas ☐
No tiene ventanas ☐
Excesivamente húmedo ☐
Ruidoso ☐
Oscuro ☐
Descripción:
____
____

¿Cómo ha cambiado su trabajo con la enfermedad?
____
____

Nivel de Ingresos

Descripción	Ingresos (S/. nuevos soles)	Descripción	Egresos (S/. nuevos soles)
Mensual	<750 ☐<br>750-1000 ☐<br>1000-1500 ☐<br>>1500 ☐	Alimentos	< 200 ☐<br>200-400 ☐<br>>400 ☐
Adicional		Vivienda	< 200 ☐<br>200-400 ☐<br>>400 ☐
Otros		Educación	< 200 ☐<br>200-400 ☐<br>>400 ☐
		Vestimenta	< 200 ☐<br>200-400 ☐<br>>400 ☐
		Salud	< 200 ☐<br>200-400 ☐<br>>400 ☐
		Recreación	< 200 ☐<br>200-400 ☐<br>>400 ☐
		Deudas	< 200 ☐<br>200-400 ☐<br>>400 ☐
		Otros	
**Total**			

V. Estado de Salud: Enfermedad actual, comorbilidades y cuidados de salud

¿Dónde cree Ud. que ha estado expuesto a la tuberculosis?
____
____

¿Ha estado Ud. rodeado por personas con tuberculosis?
____
____

¿Siempre ha vivido aquí? (localidad donde se le entrevista)
*Si la respuesta es no, saltar la pregunta.
____
____

¿Su familia siempre ha vivido aquí? (localidad donde se le entrevista)
____
____

¿Por qué se mudó aquí?
____
____
____

¿Son distintas las condiciones de vida aquí que en donde vivía anteriormente?
____
____
____

¿Cree Ud. que es más fácil contraer tuberculosis aquí que en donde vivía anteriormente?
____
____
____

Hábitos alimenticios. ¿Cuántas comidas al día come? ______________________________

¿Qué come, donde y por qué?
Desayuno: ____
Almuerzo:____
Cena: ____
Otro: ____

Síntomas de la tuberculosis

	NA	Tiempo Antes de Diagnostico	Descripción
1. Tos con Flema:	☐	__________	
2. Fiebre:	☐	__________	
3. Pérdida de peso:	☐	__________	
4. Hemoptisis:	☐	__________	
5. Otros:	☐	__________	

Ud. ha recibido un diagnóstico de:
1. Diabetes ☐ Año de Diagnóstico: __________
2. EPOC ☐ Año de Diagnóstico: __________
3. VIH/SIDA ☐ Año de Diagnóstico: __________
4. Discapacidad ☐ Año de Diagnóstico: __________
5. Otros: ____

Comportamiento de búsqueda de atención en salud

¿Cuando empezó a sentirse mal?
____
____

¿Qué es lo primero que pensó? ¿Por qué?
____
____

¿Qué síntomas hicieron que Ud. busque atención para su salud? ¿Por qué?
____
____

¿A dónde acudió Ud. primero para atender su problema de salud?
1.Farmacia ☐
2.Automedicación ☐
3.Medicina Tradicional ☐
4.Médico particular ☐
5.Profesional de salud ☐
6. Otros: ____

¿Cuáles fueron los motivos por los cuales tomó esa decisión?
____
____
____

¿Cuáles fueron las medicinas que tomó?
____
____

¿En qué fecha Ud. acudió a la visita a un médico por la enfermedad actual? (dd/mm/aaaa)
_____/_____/_____

¿Cómo se llamaba el establecimiento de salud donde acudió por primera vez?
____

¿Por qué Ud. decidió venir a este establecimiento de salud?
1.Fácil acceso ☐
2.Confianza en curarse ☐
3.Atención disponible en cualquier momento ☐
4.Referido por un personal de salud anterior ☐
5.Atención gratuita ☐
6. Aconsejado por otra persona ☐
7. Otro: _________________________________
Describa:
____
____
____

¿Cómo fue esta primera experiencia?
____
____
____

¿Cómo fueron los recursos disponibles en el centro de salud? (Por ejemplo, información sobre TB, equipos de diagnóstico) Describa.
____
____
____

¿El tratamiento que recibió del personal de salud? Describa.
____
____
____

¿Cómo fue el tiempo que tuvo que esperar para que sea atendido? Describa.
____
____

Cuando usted empezó a sentirse enfermo, ¿cuáles fueron las razones por las que Ud. espero para ir a un centro de salud?

1.Demasiado lejos ☐
2.Demasiado ocupado ☐
3.Largo tiempo de espera ☐
4.Mala experiencia ☐
5.Miedo al diagnóstico ☐
6.Negación/ocultamiento de síntomas ☐
7.Miedo al aislamiento social ☐
8.Restricciones económicas ☐
9.Mala actitud del personal del centro de salud ☐
10.Pobre calidad del servicio de salud ☐
11.Otros: ____

¿En qué fecha Ud. fue diagnosticado de TB? (dd/mm/aaa): _____/_____/_____

¿Estaba sorprendido a recibió este diagnosticado?
____
____

¿Cuántas veces Ud. busco atención médica antes de ser diagnosticado de TB?
____
____

¿A qué distancia queda el establecimiento de salud de su domicilio? (especifique tiempo)
____
____

VI. Respuesta social a la enfermedad

¿Ud. se ha sentido avergonzado por tener tuberculosis?
____
____

¿Ud. tiene que ocular su diagnóstico de tuberculosis a otras personas?
____
____

¿Ud. piensa que la tuberculosis afecta alguna de sus relaciones sociales? (incluyendo marital y familiar)?
____
____

¿Es costoso para Ud. el tratamiento de la tuberculosis? ¿Por qué?
____
____

¿La tuberculosis afecta su trabajo?
____
____

¿La tuberculosis afecta sus responsabilidades familiares?
____
____

¿Piensa Ud. que hay menos posibilidades de casarse debido al diagnóstico de tuberculosis?
____
____

¿Cree Ud. que es más difícil para las mujeres que los hombres a recibir tratamiento contra la tuberculosis?
____
____

¿Existe discriminación de las personas con tuberculosis? ¿Dónde?
____
____

¿Según tu apreciación que ayudaría a las personas a acudir más rápido después de tener los primeros síntomas de la tuberculosis al médico?
____
____

VII. Conocimientos de la enfermedad

¿Piensa Ud. que la tuberculosis es una causa de infertilidad en la mujer?
____
____

¿La TB afecta la lactancia materna?
____
____

¿La TB afecta el resultado del embarazo?
____
____

¿Ha oído anteriormente hablar sobre TB? Si ☐ No ☐

¿Antes de su diagnóstico, que has escuchado sobre TB y el tratamiento de TB?
El tratamiento es gratis ☐
El tratamiento es carro ☐
El tratamiento es largo ☐
A veces las clínicas no tienen las pastillas ☐
El tratamiento es doloroso ☐
Personas con TB recibe malo tratamiento en las clínicas ☐
Personas con TB recibe bien tratamiento en las clínicas ☐
Otro:____
____

¿Dónde obtuvo esa información?
1.Campaña de salud ☐
2.Instituciones educativas ☐
3.Amigos o familiares ☐
4.Amigos o familiares con la enfermedad ☐
5.Otro:
____
____

¿La tuberculosis es hereditaria?
____
____

¿Es contagiosa la tuberculosis?
____
____

¿Cómo se contrae la tuberculosis?
____
____

¿Cuál es la causa de tuberculosis?

¿Se puede curar la tuberculosis?

¿Existe una vacuna contra la tuberculosis?

¿Sabe cuánto tiempo durará su tratamiento?

FAMILY MEMBER INTERVIEW GUIDE

Datos Generales

Fecha de la entrevista (dd/mm/aaa): _____/_____/_____
Lugar de la entrevista: ____
Dirección de Salud: ____
Red de Salud: ____
Establecimiento de salud: ____

Edad: ____
Genero: ____
Relación a persona con TB: ____

1. ¿Que opiniones sobre la tuberculosis tienen las personas en su comunidad?

2. ¿Sí una persona tiene TB, como lo tratan los demás y que dicen sobre él?

3. ¿Cuáles son las causas que originan la tuberculosis?

4. ¿Quien tiene mayor riesgo de contraer tuberculosis?

5. ¿Cuándo una persona tiene tos con flema, fiebre o pérdida de peso que hace primero? ¿Tos con sangre?

6. ¿Es frecuente que las personas se automediquen y por qué?

7. Porque las personas esperan antes de ir al médico?

8. ¿Qué saben las personas sobre el tratamiento por TB?

9. ¿Si una persona tiene síntomas de tuberculosis, ¿qué ayudaría a la persona a acudir más rápido al médico?

FOCUS GROUP GUIDE

Fecha de Grupo Focal (dd/mm/aaa): _____/_____/_____
Lugar de la entrevista: ____
Dirección de Salud: ____
Red de Salud: ____
Establecimiento de salud: ____
Facilitador: ____
Anotador: ____

1. ¿Qué contribuye a la alta prevalencia de tuberculosis en esta comunidad?

2. ¿Quien tiene mayor riesgo a contraer tuberculosis?

3. ¿Cuáles son las percepciones de tuberculosis en esta comunidad de las personas que no tiene la enfermedad que ustedes hayan escuchado?

4. ¿Antes de ir al médico, que hacen generalmente las personas cuando tienen síntomas de tuberculosis?

5. ¿Que tipos de medicinas toman las personas antes de ir al médico? ¿Cuales son algunas razones para este comportamiento?

6. ¿Normalmente, ¿cuantos días esperan personas antes de ir al médico?

7. ¿Cuáles son algunas razones por las cuales las personas esperan antes de ir al médico?

8. ¿Normalmente, que conocen las personas sobre tuberculosis cuando ustedes los atienden por primera vez?

9. ¿Cuando las personas escuchan sobre sus diagnostica, ¿cómo reaccionan generalmente?

10. ¿Qué conocen las personas generalmente sobre el tratamiento de TB cuando conversan con ellos por primera vez?

11. ¿Como ayudan a las personas a aprender sobre su enfermedad, manejar y terminar su tratamiento?

12. ¿Cuanto tiempo esta con las afectadas por tuberculosis?

13. ¿Ustedes consideran que hay discriminación contra las personas con tuberculosis? ¿Quienes discrimina? ¿Cuáles una forma de discriminación que ustedes han observado y donde han observado estas?

14. ¿Cuáles son las consecuencias sociales por tener tuberculosis?

15. ¿Cuales son algunas de las actitudes que generalmente tiene el personal de salud que trabaja con personas con tuberculosis?

16. ¿Qué ayudaría a las personas a ir al médico más rápido cuando tienen síntomas de tuberculosis?
